# Opportunities for optimising care transitions of adults with multiple long-term conditions: a qualitative interview study

**DOI:** 10.1186/s12877-025-06264-2

**Published:** 2025-08-08

**Authors:** Stella Arakelyan, Atul Anand, Stewart W. Mercer, Nazir Lone, Marcus J. Lyall, Julie A. Jacko, Bruce Guthrie

**Affiliations:** 1 Advanced Care Research Centre, Usher Institute, University of Edinburgh, Edinburgh, UK; 2 NHS Lothian, Royal Infirmary of Edinburgh, Edinburgh, UK; 3 Centre for Cardiovascular Science, Chancellor’s Building, University of Edinburgh, Edinburgh, UK; 4 Centre for Medical Informatics, Usher Institute, University of Edinburgh, Edinburgh, UK; 5 BioQuarter - Gate, Usher Building, University of Edinburgh, 5-7, 3 Little France Rd, Edinburgh, EH16 4UX UK

**Keywords:** Transitions of care, Integrated care, Multimorbidity, Quality of care, Care coordination

## Abstract

**Background:**

The number of adults with multiple long-term conditions (MLTC) who experience frequent care transitions is rising. Improving care transitions for adults MLTC is important because transitions between and within care settings commonly lead to preventable adverse events. We explored multidisciplinary professional perspectives and experiences of managing care transitions for patients with MLTC to identify opportunities for improvement.

**Methods:**

Qualitative interviews with 30 health and social care professionals in four Scottish integrated Health and Social Care Partnerships. Data were collected between May 2023 and March 2024. Thematic analysis was used, guided by the Sustainable Integrated Chronic Care Models for Multimorbidity: Delivery, Financing, and Performance (SELFIE) framework.

**Results:**

Care transitions were described as lacking person-centredness and consistency. Variability in decisions on cross-boundary acute care pathways was largely attributed to human factors (e.g., ease of arranging referrals, a lack of trust or awareness of Hospital at Home service) by hospital specialist staff, but to clinical complexity and home environment limitations (physical and social) by community staff. Ineffective interprofessional relationships and poor communication across services were common experiences, significantly driven by a lack of integration between IT systems affecting timely access to information and by services having different priorities and pressures. Workforce shortages, knowledge gaps in managing MLTC, and long-standing capacity issues in social care were identified as important barriers to effectively managing transitions.

**Conclusions:**

We identified multiple system-level barriers to providing high-quality and safe care transitions. We proposed key improvement opportunities, highlighting the need for using system engineering and systems thinking approaches, underpinned by the active engagement of patients, carers, professionals, and wider stakeholders to drive meaningful and sustainable change in transitions of care.

**Supplementary Information:**

The online version contains supplementary material available at 10.1186/s12877-025-06264-2.

## Introduction

Health and social care systems are increasingly challenged by the growing number of adults living with multiple long-term conditions (MLTC) who experience frequent care transitions and are at increased risk of poor outcomes [1, 2]. Care transitions are transfers of care which may be between care settings (e.g., from home to hospital, hospital to care home) or within care settings (e.g., moving wards within the hospital) [3] and are key points of failure in systems which are disease-centric, fragmented, and/or poorly coordinated. Health and social care policies prioritising shorter hospital stays and quicker return to home care [4] risk leaving patients/families unprepared to navigate the complexities of care transitions [5]. Evidence shows that care plans are often incompletely implemented following transitions due to poor communication and lack of care continuity (i.e., consistent and coordinated care delivery), increasing patients’ risks of adverse events (e.g., falls, emergency department (ED) reattendances, hospital (re)admissions, and death) [6, 7]. An estimated 20% of patients experience an adverse event following a transition from hospital to home [8], and early ED reattendance rates are rising, reaching 15% of all adults within 30 days of transitioning from hospital to community care settings [9, 10].

With population ageing and increasing MLTC prevalence, more effective management or optimisation of care transitions is essential [11]. Better integration of health and social care services has long been advocated as a system response to achieve this [11], but integration in many countries is challenged by separate budgets, silo mentalities, varying priorities, and issues with interprofessional communication and collaboration across boundaries [12]. Evidence for the effectiveness of integrated and transitional care models in improving outcomes for adults living with MLTC is inconclusive [13–15], and there remain uncertainties on how best to promote continuity of care and improve the quality and safety of care transitions for adults living with MLTC.

High-quality and safe transitions are person-centred, well-planned, and coordinated care processes that are tailored to the unique needs and preferences of patients/families, ensure seamless and accurate information exchange across multidisciplinary care teams, and include timely follow-up to mitigate risks of adverse events [7]. Previous research explored potential strategies for optimising care transitions for older adults living with complex needs in England [5, 16] and internationally [17–19]; however, evidence specific to MLTC in the Scottish context is lacking. Our initial patient and public involvement (PPI) work emphasised that care transitions are experienced in a complex and interconnected system. PPI contributors stressed the need for system-wide and context-specific improvements to optimise care transitions, rather than focusing solely on a single transition or localised processes, shaping the broad scope of our study. This study, therefore, aimed to explore multidisciplinary and system-wide professional perspectives and experiences of managing care transitions for adults living with MLTC to identify opportunities for improvement.

We sought to address the following research questions:

- What challenges are faced by multidisciplinary professionals in managing care transitions for adults living with MLTC?
- What approaches do multidisciplinary professionals use to navigate the complexities of care transitions for adults with MLTC?
- What system-wide improvements do multidisciplinary professionals identify as central for optimising care transitions for adults living with MLTC?

## Methods

### Study design and setting

The study used qualitative analysis of semi-structured interviews with a purposive sample of health and social care professionals. The setting was four Health and Social Care Partnerships (HSCPs; one city and three mixed urban/rural) responsible for integrated care delivery in a large Scottish Health Board serving ~ 900,000 people. Integrated care brings together key aspects in the design and delivery of care systems to address care fragmentation. In Scotland, the integration of health and social services was legislated in 2016 [20], creating 31 integrated HSCPs which are usually coterminous with the 32 Scottish local authorities, with the larger of the 14 National Health Service (NHS) Health Boards having several HSCPs within them [21]. Health boards and local authorities pool a proportion of their budgets and devolve budgetary control to HSCPs, which deliver and organise primary health care, community health care and adult social care. Primary health care is provided by general practices free at the point of care, with General Practitioners (GPs) acting as gatekeepers for other health services [22]. General practices work in clusters of 8–10 practices, which have a dual role of improving care quality and providing leadership in care integration. Acute hospital care is free at the point of care, and access can be initiated by patients/carers (self-referral), GPs, or paramedics, with the intention of discharge from hospital when medically fit (stable) [23]. Adult social care operates on a means-tested basis, with a mix of public funding, personal contributions, and largely private sector provision [21].

### Patient and public involvement

This study was part of the Artificial Intelligence and Multimorbidity: Clustering in Individuals, Space and Clinical Context (AIM-CISC) programme, which involved PPI contributors (adults living with MLTC, carers, professionals) throughout the entire programme to support the research aims. Early discussions with PPI contributors about the planned work on optimising care transitions confirmed support for the proposed methods. Contributors, however, emphasised that care transitions are experienced in a complex and often fragmented health and social care system, highlighting the importance of addressing system-wide and contextual challenges rather than focusing on a single transition or improving discrete localised processes. Their input informed the broader framing of our study and shaped our approach to identifying improvement opportunities that align with the principles of safe and high-quality care transitions.

### Sampling and data collection

Sampling was purposive to recruit a heterogeneous sample of professionals with key roles in planning and/or managing care transitions for adults living with MLTC across community health and social care and hospital services. Eligible professionals were identified from public sources and our professional networks, with initial contacts made electronically. Professionals who agreed to take part were interviewed at their workplace or online using video-conferencing (MS Teams) by a female research fellow with a doctoral degree (SA). The participants were asked to nominate eligible professionals with complementary experience to contact after the interview.

Interview data were collected between May 2023 and March 2024. The interview topic guide included questions on experience with integrated care provision, multidisciplinary working and coordination, and continuity of care; approaches used to ensure person-centredness and shared decision-making in care; experiences with adjustments used in clinical work to support optimal care during transitions; and potential solutions to improving care in their current clinical context and across the whole system (Additional file 1). The guide was pilot-tested with two participants, and all subsequent participants were asked the same set of core questions. Interviews lasted between 40 and 75 minutes and were audio-recorded. The recruitment, data collection, and analysis occurred iteratively, and we continued recruiting until we reached a point of theoretical saturation [24].

### Ethics approval and consent to participate

The study adhered to the Declaration of Helsinki (https://www.wma.net/policies-post/wma-declaration-of-helsinki/) and was approved by the North West - Greater Manchester West Research Ethics Committee (REC reference: 22/NW/0313). Participants were sent information about the study and returned signed consent forms before interviews if they were willing to participate. All professionals were assured of confidentiality and anonymity and informed about their rights to withdraw at any point.

### Analysis

The interviews were audio-recorded, transcribed verbatim, and imported to NVivo v12 software for coding (QSR International Pty Ltd., 2018). Data were analysed using an inductive-deductive (abductive) approach [25] that was theoretically guided by the SELFIE (Sustainable Integrated Chronic Care Models for Multi-Morbidity Delivery, Financing and Performance) framework for integrated care in multimorbidity [26]. After familiarisation with all interview transcripts, one researcher (SA) inductively coded five transcripts and developed the initial coding tree, comprising 12 parent and 36 child codes. Through analytical discussions with a senior researcher (BG), the codes were thematically grouped and mapped onto the contextual domains and conceptual categories of the SELFIE framework. We used SELFIE as an analytical tool because it provides a comprehensive, structured, and person-centred approach to understanding and improving care for adults living with MLTC. More specifically, it places adults living with MLTC and their environment at its core, with integrated care concepts structured across micro-, meso-, and macro-level domains of Service Delivery, Leadership and Governance, Workforce, Financing, Technologies and Medical Products, and Information and Research [26]. In the context of our work, SELFIE offered a holistic lens for examining how various components of care interact to influence care at the community-hospital interface. The final coding framework was peer-debriefed with the research team and deductively applied to all transcripts. Throughout the analysis, themes were critically evaluated through constant comparison of coded data within and across themes to identify emerging patterns and relationships.

The research team comprises health services researchers and clinicians with expertise in qualitative methods, and we used several strategies to enhance the trustworthiness of the findings [27]. To ensure transparency in data analysis and reporting, we used processes allowing for an audit trail of data collection, management, and analysis. We cross-checked transcripts for the accuracy of representations of interview discussions with two professionals, but no feedback was sought on the findings. Our reporting of findings followed the Consolidated Criteria for Reporting Qualitative Research (COREQ) guidance [28].

### Results

We approached 46 health and social care professionals, of whom 30 participated. Twenty-two participants were women, and most had over 15 years of working experience (mean 21 years, range 6–39) and mean eight years of experience in their current role (range 1–25 years). Eight were employed as managers, and six performed senior roles across community and hospital services (Table 1).

**Table 1:** Participant characteristics

Characteristic	Total number
Age (year)	
20–29	2
30–39	10
40–49	8
50–59	10
Gender	
Female	22
Male	8
Setting	
Community (primary care, community clinics, HSCPs)	18
Hospital	12
Professional Role	
Older People’s Community Service Manager	2
Clinical Pharmacy Service Manager	1
Care Home Manager or Deputy Manager	3
Community Link Worker or Service Manager	2
Hospital Clinical Nurse Manager	1
Consultant Geriatrician	2
General Medicine or Acute Care Consultant	3
Old Age Psychiatrist	1
Hospital Advanced Nurse Practitioner	2
General Practitioner	4
Out-of-hours General Practitioner	1
Allied Health Professional*	4
Clinical pharmacist	2
Social Worker	2
Years of experience since qualified	
< 5	-
5–9	3
10–14	5
≥ 15	22
Years of experience in current role	
< 5	12
5–9	11
10–14	2
≥ 15	5

HSCP– Health and Social Care Partnership

*Physiotherapist 2, Occupational therapist 2

**Fig. 1:**
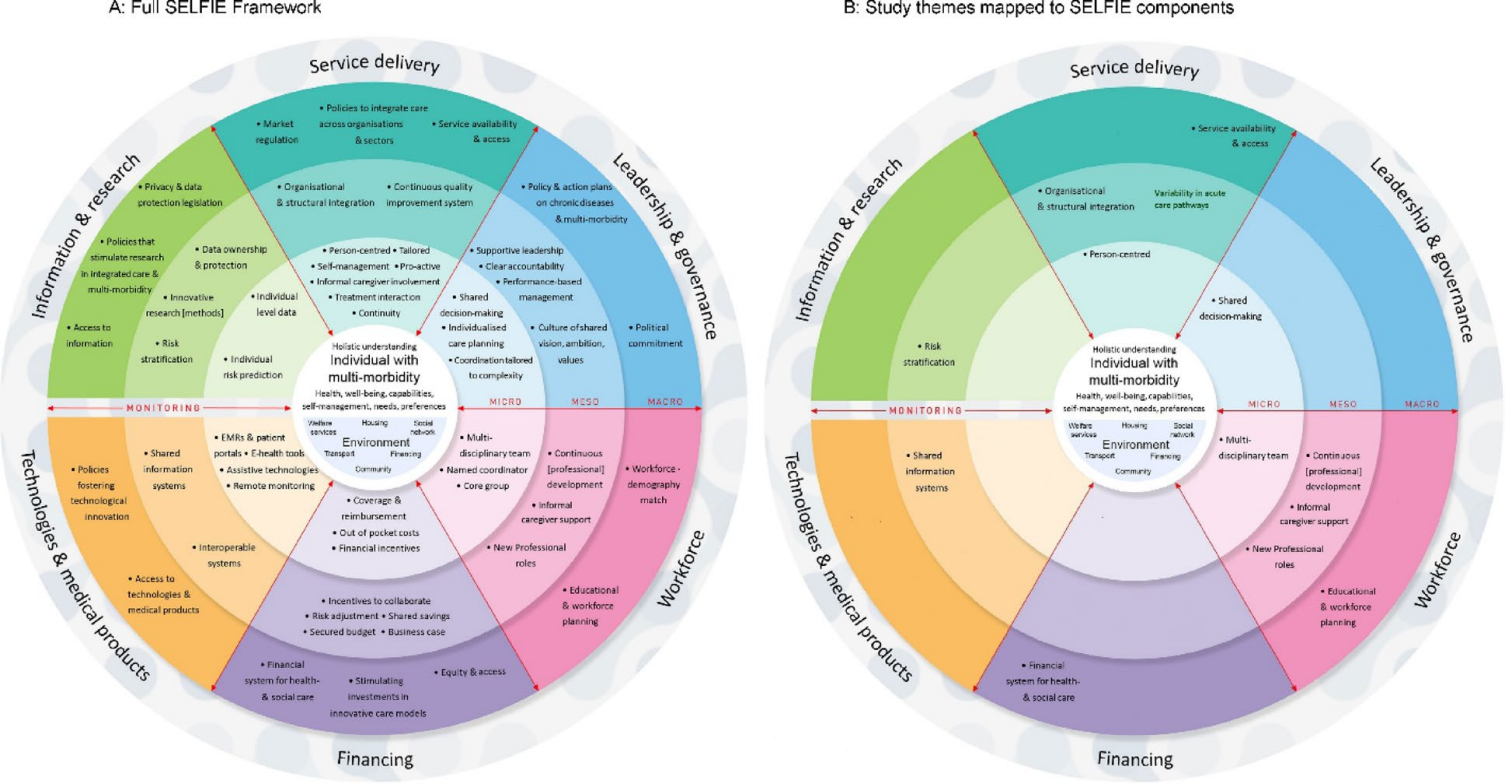
SELFIE framework domains and components. Reproduced from Leijten et al. [26] SELFIE is an Open Access framework distributed in accordance with the terms of the Creative Commons Attribution Non-Commercial No Derivatives (CC BY-NC-ND 4)

We present the results according to micro-, meso-, and macro-levels of influence mapped to six SELFIE domains (where present in the data) and corresponding concepts (Fig. 1). Additional quotes are provided in Additional file 2.

### Micro-level service delivery

#### Person-centred care

Hospital doctors described transitions of care for adults living with MLTC as lacking person-centredness and consistency. They felt that many inappropriate acute referrals and admissions could have been avoided if community care teams were more effective in respecting and enabling patients’ documented wishes and priorities.

*“We still see a number of patients who are admitted into the hospital who have got a key information summary that says that their wishes are to not be admitted to hospital.” [Consultant Geriatrician]*.

They also felt that acute referrals were often inappropriate for complex patients (who often lacked the capacity to advocate for themselves) and that poor communication among community care teams resulted in default front-door referrals. However, an Older People’s Community Service Manager described hospital teams’ perceptions of inappropriate acute referrals as framed by them *“lack[ing] understanding of how community services operate and do not know any of the circumstances beyond the person that is in front of them; it is not in their orbit*”. One hospital doctor noted interprofessional conflict when community professionals make acute referral decisions against patients’ wishes.

*“I had experiences […] where we got community physiotherapists in that sort of situation to go and assess patients and see if maybe some equipment might prevent their falls. The therapist took one look at the patient and said: ‘I’m not seeing them because they need to go to hospital.’ That’s not patient-centred*,* the patient doesn’t want to go to the hospital. We didn’t ask whether or not the patient needed to go to the hospital*,* we asked to try and make things better for them.” [General Medicine Consultant]*.

Hospital doctors also described patient movements within the hospital as *“chaotic”*, with multiple barriers to early discharge. High demand for geriatric services commonly exceeded bed capacity, resulting in patients being *“periodically boarded out*” to less appropriate wards with perceived worse outcomes as a result (longer hospital stays, functional deterioration, confusion).

*“Patients come in through ED and then go to a bed in the Acute Medical Admissions Unit. They might be boarded to a non-Medicine of the Elderly (MoE) ward and then repatriated to MoE but they may not.… If you think about the number of patient moves that happen*,* and we know that when we board our patients their outcomes are worse. So that is not a person-centred pathway.” [Consultant Geriatrician]*.

### Micro-level leadership and governance

#### Shared decision making

Community care teams talked about the complex interplay of factors contributing to decision-making on acute referral pathways, with shared decision-making and holistic understanding of patients within their social and physical environment being core to care in this context.

*“Shared-decision making is absolutely the bread and butter of what GPs do…We are much better and less paternalistic than secondary care*,* and we are good at gauging what people can manage. A lot of that comes from the sheer volume of patients that we see in their homes and getting that hands-on experience of what day-to-day life is like for somebody. And that discussion with the patient informs to what extent we encourage someone to take one or another course of action.” [General Practitioner].*

In contrast, a hospital specialist noted a significant power imbalance between doctors and patients in the hospital setting, emphasising how a busy environment and differences in appearance (doctors professionally dressed during ward rounds, and patients in pyjamas and confined to bed) create challenging conditions for shared decision-making.

### Micro-level workforce

#### Multidisciplinary teams

Healthcare professionals highlighted the benefits of multidisciplinary team (MDT) working in delivering safe and effective care. A hospital social worker noted the advantages of daily meetings with the NHS MDT in identifying patients ready for discharge and ensuring continuity of care. However, in emergency settings, MDT working was occasionally a source of delay.

*“I think in some ways*,* having more allied health professionals involved is a great thing and it’s what we need*,* but just occasionally in the ED*,* it does slow things up because we then do call them and it takes time for them to come back. We could probably just deal with it ourselves.” [Acute Care Consultant]*.

Further, a community healthcare professional described communication issues and a lack of clarity and understanding of various teams’ roles and processes involved in patient handovers.

*“Multidisciplinary care is good but it’s very difficult to understand what each team does and then how patients move from one team to another. I think sometimes those transitions [from hospital to home] and touchpoints between multiple teams looking after patients are not always good.” [Older People’s Community Service Manager]*.

### Meso-level service delivery

#### Variability in cross-boundary acute care pathways

Hospital specialists noted inconsistency in care pathways for adults living with MLTC, which was largely attributed to ‘human factors’ ─ a lack of awareness or trust in community urgent services (e.g., Hospital at Home) and/or admissions being easier to organise than the community alternatives.

*“I think there’s a whole range of reasons within that [variability in referrals]. I think some are human factors*,* some of the fact that actually*,* it’s very easy to admit someone to hospital. Because all they do is make one phone call and speak to a telephone advisor and they organise the ambulance*,* and the GP goes on to the next home visit.” [Consultant Geriatrician]*.

Community care teams noted that decisions on acute referrals commonly involved trade-offs between safety and efficient use of services. GPs often assessed the benefits of hospital care in the context of worsening acute response times - *“waiting for 6–8 hours for an ambulance to take them [patients] to the medical ward where they have a further wait to be seen”* - against the risks of keeping patients in their homes, with a higher threshold for referral set up for patients with less complexity.

*“The people that I refer to hospital are more likely to be frail*,* elderly*,* have multiple morbidities because the risk of them becoming more unwell at home is higher” [Out-of-hours General Practitioner]*.

#### Organisational and structural integration

Longstanding challenges in communication and information flow breakdowns across services were widely recognised. Hospital specialists talked about ineffective communication and relationships across community services as a key factor contributing to increased patient flow to acute hospital services. In contrast, community healthcare professionals talked about information flow issues at the community and hospital interface, compromising holistic care. Lack of clarity in discharge letters on practical matters, e.g., *“what antibiotics patients got*,* when did they get it*,* and at what dosage”* often made care delivery for early supported discharged patients challenging.

*“We did a survey to ask GPs what were the barriers to the flow of patients*,* and one of the things that came out of it is that if there was better quality communication and you felt you had more of an understanding of what was going on with your patient*,* you might then feel more able to take back their care and look after them more holistically.” [General Practitioner]*.

Care home managers talked at length about suboptimal experiences with information flow when care home residents were admitted and discharged from the hospital. Hospital discharge letters often missed critical information, putting care home residents at risk of harm.

*“The big thing is communication. When somebody goes into hospital*,* we give them quite a holistic view of what that person’s needs are. Quite often that goes into the admissions unit or A&E*,* and then each ward phones us and asks us all those questions again because it’s not been passed on…It goes nowhere.” [Care Home Manager]*.

Hospital specialists recognised variability in communication and issuing of discharge letters in a timely fashion. This was largely attributed to overstretched service capacity and inadequate junior doctor and nurse staffing levels.

*“During times of acuity*,* as many as half the patients sometimes do not get the immediate discharge letter as to what’s happened during this acute admission.…With regards to the final discharge letter*,* sometimes it takes three months… So*,* the processes are under strain because of the acuity or lack of staff.” [General Medicine Consultant]*.

To speed up information flow across services, other modes of communication such as phone calls and e-mail were sometimes used, but these workarounds further increased demands on staff time. There was a shared sentiment across the majority of participants that communication and collaboration across services and organisations were ineffective.

*We need better relationships between the hospital and the community and the hospital and other outreach services. Often the patient will have various contact numbers and various people to help in the community but it takes us a long time to find out who these people are and how to contact them.” [Hospital Clinical Nurse Manager]*.

### Meso-level workforce

#### Continuous professional development

Healthcare professionals noted that training of the hospital workforce is largely specialism/setting specific, leaving professionals less well prepared to cater to the complex needs of patients with MLTC.

*“People who look after patients at the front door and A&E physicians are very used to dealing with acute emergencies but dealing with the interactions of different diseases and multimorbidity is less their specialty.” [General Medicine Consultant]*.

Care home managers talked about an acute need to raise awareness, skills, and competencies of care home staff in looking after adults living with MLTC and polypharmacy, emphasising that training programmes should be organised and delivered by the NHS to both public and private care homes. There was a sentiment across all professionals that enhancing generalist and polypharmacy review (deprescribing) skills across the whole health sector could improve the management of MLTC.

*“I think training could help [improve care]*,* and I think it’s not just doctors*,* is it*,* it’s nurses and the rest of the allied health professionals as well.” [Old Age Psychiatrist]*.

However, care home staff also commented that healthcare professionals did not always recognise their skills and competence which hindered collaborative working.

*“Important that professionals see that although we are carers*,* we do have the knowledge and know our residents and for them to trust that we can give our judgment and for it to be taken seriously. We have nurses that come in and sometimes they really talk down toward the staff… and it’s just that kind of attitude is quite off-putting” [Care Home Deputy Manager].*

#### New professional roles

Hospital specialists noted insufficient skills in managing MLTC and frailty at the hospital front-door, with a local response being the development of a front-door frailty service. For patients requiring admission, capturing key information about their social circumstances and care options was deemed critical. To improve hospital-to-home transitions for complex patients, a complex discharge planner role had been piloted. In the community, new *‘integrated roles’* had been created to improve care continuity at the hospital and community interface.

*“One of the innovations is integrated pharmacists who are looking at all the discharges from hospital to reconcile the changes that happen in hospital with patients’ ongoing prescriptions in primary care. That’s because the IT systems don’t talk to each other.” [General Medicine Consultant]*.

A community professional stressed that some patients would benefit from a care manager or coordinator to navigate services, improve inter-professional communication, and manage home visits, care options, and legal matters.

#### Informal caregiver support

Healthcare providers often leant on family support to expedite the discharge of patients with MLTC, for example by proactively negotiating with families to informally provide care until social care packages were in place. However, despite many families taking long-standing caregiving roles, there was a lack of targeted caregiver support, resulting in carer stress and burnout.

*“So*,* I’m also the lead for unpaid carers*,* and a story that is shared time again [is that] they’re absolutely on their knees.” [Older People’s Community Service Manager]*.

### Meso-level technologies

#### Shared information systems

Healthcare professionals noted recent improvements in accessing some patient data from other parts of the system (e.g., test results, access to community teams’ notes). However, such access was only ever partial and often difficult in practice, leading to inefficiencies in obtaining up-to-date information for care planning.

*“Our complex discharge planner wasn’t able to contact social workers*,* leaving messages and they weren’t getting back to her because they were busy… It would be sometimes days before she got an answer to the sort of question that she could have looked up on their notes.” [Hospital Clinical Nurse Manager]*.

Having access to reliable and up-to-date information on patients’ MLTC and treatment was deemed central to improve care quality and safety. An out-of-hours GP noted issues with accessing information about acutely ill patients’ medical history and prescriptions during home visits where internet and electronic health record access is restricted. Care home managers similarly highlighted issues with accessing information held in NHS systems which often compromised holistic and safe care delivery.

*“The thing that doesn’t work well for us is not having access to the information we need. The NHS staff that come into the home have access to a big database that has everything about a person on it. We get a snapshot. Some families don’t know everything about their loved ones’ healthcare. Some people don’t have families*,* so quite often we don’t find out things until the last minute.” [Care Home Manager].*

### Meso-level information and research

#### Risk stratification using routinely collected data

Healthcare professionals discussed the potential of electronic health records and artificial intelligence to develop tools for early identification of patients at risk of adverse events (e.g., falls, delirium, sepsis, collapse) for early targeting. A hospital doctor emphasised the need for tools to identify patients who can be safely managed in the community rather than admitted, but also the need to ensure that the right services were in place to deliver that.

*“For people who aren’t so complex*,* frail*,* and unpredictable*,* a risk prediction tool that might help to work out which patients could relatively safely not be admitted to the hospital and be looked after at home will be useful. And I think the use will be trying to establish where that person can be looked after*,* in what kind of care environment they can be looked after*,* and what their journey would be.” [Consultant Geriatrician]*.

The hospital doctor noted the significance of accurately predicting hospitalisation risks and life expectancy as essential components in advancing the practice of realistic medicine [29].

### Macro-level service delivery

#### Service availability & access

Healthcare professionals noted that a lack of resources to meet social care needs in the community often contributed to inappropriately delayed discharges for otherwise medically fit patients.

*“All of the time that I was working in hospital*,* I could have discharged about 50% of patients sooner if adequate care packages and access to social care were available. The extended stays weren’t due to risk aversion or indecision*,* but rather a significant gap between patients’ needs and the available community care options and resources”. [General Practitioner]*

A social worker recognised social care packages took time to set up, despite priority always being given to people being discharged from hospital and intermediate care, and noted that patients from rural areas, in particular, waited much longer for social care.

*“People who are in a more remote area will wait longer*,* but we have seen people waiting around a year for packages of care. We always try to prioritise hospital discharges and [intermediate care home name] discharges*,* but there are people who have been waiting an excess of between 3–6 months. But*,* again*,* that is all dependent on their area because there are some places that the care agency doesn’t have the staff to reach.” [Social worker]*.

### Macro-level workforce

#### Educational and workforce planning

Healthcare professionals highlighted a need for development of the workforce to provide needs-based care for patients through more generalist approaches.

*“Patients tell us that they want to be seen holistically*,* they want their needs to be dealt with holistically*,* and they still face big challenges because training and services are delivered by body area or a particular pathology.” [Community Allied Health Professional].*

Further, workforce recruitment and retention problems were noted across services, compromising integrated ways of working and care quality.

*“A barrier [to good care] is the retention of staff in general. So you just don’t have enough people around [which] means that there are not enough people able to work in an integrated team.” [General Medicine Consultant]*.

A social worker talked about staff shortages (social workers, care assistants, OTs), delaying the initiation of support packages for older people with complex needs in the community. Similarly, care home managers highlighted longstanding and worsening recruitment and retention challenges.

*“We recruit new staff*,* we train them and one day they say*,* “Financially*,* it’s better to work somewhere else.”*,* and they leave… Staff groups changed*,* most are from African countries and many of them are studying and I’m worried that they will finish university and disappear. So*,* it’s very important to attract people who are staying in the care sector and better pay would help.” [Care Home Manager]*.

### Macro-level financing

#### Financial systems for health and social care

Healthcare professionals noted that hospital-centric service models and chronic underfunding and under-resourcing of community services played a critical role in inappropriately delayed discharges and poor integration of services. They raised the importance of shifting funding from hospitals to the community and enhancing social care capacity to ease pressure on acute services. Private care home managers talked about the lack of government funding to allow the expansion of capacity for publicly funded care home residents, despite the ability of the sector to expand if need be.

*“We’re always hearing that there are people in hospital who are bed blocking and needing care. We have the capacity and I don’t know if it’s a problem with social work.” [Care Home Deputy Manager]*.

Participants routinely highlighted macro-level financial and other pressures negatively impacting the recruitment and retention of staff, including Brexit, visa regulations, and pay restraint.

## Discussion

Participants identified micro-, meso-, and macro-system level barriers to implementing high-quality and safe care transitions for adults living with MLTC, which mapped predominantly to the Service Delivery and Workforce components of the SELFIE framework (Fig. 1), although with additional substantial discussion of shared information systems. At micro-level, care transitions were perceived by participants as pathways that lacked person-centredness and consistency, although the problems emphasised depended on whether the participant was working in the hospital or the community. Participants in all settings recognised that care often involved trade-offs between quality and safety and the need to rapidly manage high workload– the ‘efficiency-thoroughness trade-off’ [30, 31]. At meso-level, most healthcare professionals noted that inefficient communication and poor inter-service relationships compromised efficient information flow, resulting in unnecessary hospital referrals and admissions, delayed discharges, and worse patient outcomes. At the macro-level, there was consensus that community healthcare and social care were chronically under-resourced, and that shifting care out of the hospital into the community was critical to optimising use of hospital beds. Participants provided limited insights on meso- and macro-level influences for Leadership and Governance, Technologies, and Information and Research domains of SELFIE. These levels of influence may be less visible to participants, or perceived as beyond their control, leading to acceptance of the *status quo*.

Previous qualitative research on care transitions of adults living with MLTC from Denmark [32] and England [33, 34] and transitions for older adults [35] reported similar challenges to continuity and care coordination. Møller et al. [32] report that by *“building bridges”* between services working in silos, nursing staff were able to better manage complex patient care transitions, suggesting that external coordination across hospitals and local authorities and internal inter-professional teamwork is essential and should be strengthened for safer transitions. Baxter et al. [33] suggest that having a good knowledge of the patient, building trust and rapport across disciplinary silos, and bridging gaps in the system by enhancing communication and adapting to evolving services and competing priorities is essential for delivering *“exceptionally safe”* transitions, but the implementation of these is a challenge. Hedqvist et al. [17] further illustrate that inter-organisational collaboration spans from ‘simple division’ to ‘full alliance’, contributing respectively to either care fragmentation or integration. They propose that care coordination for complex patients could be improved by establishing robust communication mechanisms, utilising digital technologies for efficient information exchange, clearly defining professional roles and responsibilities, and ensuring that health policies are aligned with clinical practice. Our findings are also supported by others, suggesting that a conducive environment that promotes good inter-professional relationships and trust is essential to MDT working and tailored care planning during transitions [12, 32, 33]. To facilitate this, proximity and informal interactions are central for sharing experiential knowledge and building mutual understanding and trust [36]. Further, similar to us, Coombs et al. [37] found that safe transitions are achievable if there is enough capacity in the community and social care services to deliver care, but decisions need to be informed by patient/family choice after open communication of potential risks and mitigation strategies [38].

Strengths of the study include the purposive recruitment of a diverse range of health and social care professionals across the system, and analysis mapped to the SELFIE framework, which ensured that the findings were systematically mapped across multilevel system domains and policy areas, highlighting key areas for system-wide improvements. Limitations include the broad scope of the study informed by our PPI contributors and participant recruitment, which was limited to a single large health board. Therefore, the transferability of findings to other contexts needs to be carefully considered. However, other studies have found similar results, and the identified challenges are likely to be common to many areas in the UK and internationally. Further, only professional perspectives were examined. While understanding the experience of patients and carers is also critical to system improvement, professional perspectives are important to understand in their own right. While the SELFIE framework offered a valuable analytical lens, its application within the UK’s fragmented health and social care system posed challenges, particularly in capturing the dynamic interactions across system levels. Its emphasis on structural and policy-level factors may have overlooked critical aspects of professional experiences, including organisational climate, institutional norms, and the adaptive workarounds employed in daily clinical practice.

### Implications for policy, practice and research

Healthcare professionals identified multiple challenges to implementing high-quality and safe care across multiple transition points at the community and hospital interface. Addressing these will require multi-level changes within the system, centred around how services are organised and resourced. At micro-level, holistically assessing patients’ needs, discussing and documenting patients’ wishes, health goals, expectations and those of family members will help create shared patient knowledge across care teams, reducing reactive approaches to care and minimising risks during patient handovers. Hospital acute services will benefit from increased MDT working and better training in managing MLTC and linked mental health and social complexities.

At the meso-level, expanded provision of urgent community response and Hospital at Home teams may improve the quality and safety of care at home and minimise admission, and the provision of bridging higher intensity community services may better support discharged patients awaiting long-term social care packages in the community. Creating an enabling environment for improving collaboration, effective teamwork, and information exchange across boundaries will help improve continuity and safety of transitions. Applying new approaches to prediction in routinely collected data can be used to better identify patients who can be safely managed in the community, thereby avoiding unnecessary hospital admissions. Improving needs-based support for informal carers will be essential to sustain the effectiveness of formal care services.

At the macro-level, better integration between health and social care is essential, and targeted investment and resourcing across the whole system are needed. Workforce planning and policies to improve recruitment and retention in both health and social care are critical. The NHS Long Term Workforce Plan has committed to training more staff, boosting staff productivity and retaining existing staff by offering career support, flexible working conditions, and improving organisational culture [39], but its delivery remains uncertain. Finally, revamping healthcare education by promoting the learning of more generalist skills and inter-professional collaborative practice will make professionals better prepared to face the long-term and complex care needs of the ageing population.

Although we propose potential opportunities for improving care transitions for adults living with MLTC, the implementation of these within complex adaptive health and social care systems remains uncertain. This is because health and social care systems are embedded within broader social, political, and economic contexts, where dynamic interactions both within the systems and wider contexts are inherently complex, nonlinear, and continuously evolving in unpredictable ways. One approach in implementation is to focus on improving a single transition (e.g., discharge from hospital to home [40]), but such transitions are only one step in more complex pathways and systems, and the limitations of this were stressed in our PPI work. Another approach is using system engineering and systems thinking techniques (e.g., soft system methodology, process modelling) to system redesign [41]. These techniques focus on people and understanding of underlying relationships and characteristics of working system components, design and risk management [42]. We believe further research using system engineering, systems thinking and systems performance measurement techniques is needed to examine and address the complexities and challenges that emerge when making major changes to single parts (e.g., discharge to home or care home) of a very complex adaptive system. Such system approaches and techniques will also require involvement of patients, carers, professionals and wider stakeholders in collaborative co-design to maximise the effectiveness of care transitions improvement interventions while minimising unintended consequences across the system as a whole.

## Supplementary Information

Below is the link to the electronic supplementary material.

Supplementary Material 1

Supplementary Material 2

## Data Availability

The available data includes verbatim quotes taken from excerpts of the transcripts, which are presented in this paper. Anonymised transcripts could be made available to other qualified researchers upon reasonable request to the authors and only with explicit permission from individual participants.
