# Supplementary material for: Opportunities for optimising care transitions of adults with multiple long-term conditions: a qualitative interview study
Source: BMC Geriatr. 2025 Aug 8;25:607. doi: 10.1186/s12877-025-06264-2 (PMC12333269; doi:10.1186/s12877-025-06264-2)
Supplement: Supplementary file 1 — Supplementary Material 1 [file 12877_2025_6264_MOESM1_ESM.docx]

**Additional file 1. Interview topic guide**

With an ageing population, better care for people with multiple long-term conditions (or multimorbidity) is one of the major challenges faced by the NHS and social care services. Your experience and expertise are critical to helping us understand existing care pathways for people with multiple long-term conditions and to identify potential interventions and better ways of organising care.

- Can you briefly tell me about your current and past clinical roles, particularly in terms of looking after people with multiple long-term conditions?
- What elements of care work well for people with multiple long-term conditions? What elements of care don’t work so well?
- How would you improve care for people with multiple long-term conditions in your current workplace?
  - What would it take to make this kind of change happen?
- How would you improve care for people with multiple long-term conditions across the system, key boundaries and transitions (transitions within hospital, hospital to community, community to hospital, within community)?
  - What would it take to make this kind of change happen?
- What approaches do you use to ensure information continuity across services? Prompt for written communication or other ways of communicating (emailing, phone or video calls) across services.
  - When are they likely to be helpful or critical?
- What approaches do you use to ensure management continuity across services? Depending on the earlier responses, prompt for care coordination (who does it), multidisciplinary team assessment or comprehensive geriatric assessment (who is involved, what is the impact on time-workload pressure), frailty team or similar, care pathways or guidelines.
- What approaches do you use to ensure shared decision-making in care?
- How do you encourage and support people with long-term conditions to self-manage their own health? Prompt for approaches to support informal carers and family members in facilitating patient self-management, if not mentioned.

We have come to the end of our interview. We have talked about many things.

- In your opinion, is there anything important that we have not addressed?
- Do you have anything to add in relation to today’s interview?
