# Supplementary material for: Opportunities for optimising care transitions of adults with multiple long-term conditions: a qualitative interview study
Source: BMC Geriatr. 2025 Aug 8;25:607. doi: 10.1186/s12877-025-06264-2 (PMC12333269; doi:10.1186/s12877-025-06264-2)
Supplement: Supplementary file 2 — Supplementary Material 2 [file 12877_2025_6264_MOESM2_ESM.docx]

**Additional file 2. SELFIE components, sub-themes and illustrative quotes at micro-, meso-, macro-levels**

| **MICRO LEVEL** | |
| --- | --- |
| **Service delivery**  *Person-centred care* | “To make pathways person-centred, I think it is about finite coordination and making sure that people are matched or directed to the right service and support they want…We should enhance long-term conditions specialists and practices that would actually enhance the experience of care for the individuals. The link with acute hospitals is important as hospitals hold on to frail people because they are not sure how to get that individual the right support when they are ready and want to leave hospital.” [Older People’s Community Service Manager] |
| **Leadership & governance**  Shared decision making | “I think in hospital there is a big power difference between doctors and patients. We are in a position of power, and we are there on our ward rounds dressed in work clothes and patients lying down in their pyjamas with their hair dishevelled. It isn't a good, conducive environment for shared decision-making. I try very hard to do that, and there's also the time pressures, particularly in the acute admission unit. It makes it really challenging, and often, you have the conversations that you have to have rather than the ones you want to have. [General Medicine Consultant] |
| **Workforce**  *Multidisciplinary teams* | It's good that more staff are involved in patient care discussions. We recently introduced length-of-stay meetings. These meetings focus on complex patients who have multiple comorbidities and are frail, and we bring together a diverse team, including OTs, Physios, Consultants, and discharge hub staff, to identify medically fit patients and determine what needs to be done for their safe discharge. [Hospital Advanced Nurse Practitioner] |
| **MESO LEVEL** | |
| **Service delivery**  *Variability in cross-boundary acute care pathways* | “I work in a Hospital at Home, and 25-30% of patients have heart failure. So there's a very large proportion of our patients who are going through the Hospital at Home and never touching secondary care. But equally, there would be heart failure patients that go into second care and get managed by cardiologists and have a length of stay variability of 5 to 15 days. So what's happening there in terms of why are some coming to hospital, and some are going into secondary care? The answer is that we are variable, the referrers are variable.” [Consultant Geriatrician]  “It's sometimes frustrating when we know that there are patients in the acute hospital who don't need and probably don't want to be there…Lots of my colleagues are on board and refer to Hospital at Home service, but there's a significant proportion of people who just don't believe in it as a model of care; it is not on their radar.” [General Medicine Consultant] |
| *Organisational and structural integration* | “The first thing I'd say is there is a problem with communication, and it’s not just primary care; it is the Scottish Ambulance Service, it’s care agencies. It’s not clear to everybody involved in looking after a patient what the expectation is when they become unwell and the default is to call an ambulance and come back to hospital.” [Consultant Geriatrician]  “So, although they say there’s health and social care integration unless I phone my social work colleagues who are already really busy and overstretched….so patients in hospital are not their priority. Their priorities are the patients who are in the community who have potentially lots of needs. And we have to ask them to look it up for an update of information for us.” [Hospital Advanced Nurse Practitioner]  “Sometimes it can feel like we are fighting against each other [social care] and I don’t think that’s productive. I think we need to work together as well to try and sort this delay in packages out”. [Hospital Clinical Nurse Manager]  “It's mainly calls that we end up chasing people, even just between the GP and the pharmacy and between us. It's a lot of the responsibility falls on us to keep chasing people and it's a lot of work”. [Care Home Deputy Manager] |
| **Workforce**  *Continuous professional development* | “There is a need for training and education in terms of medication reviews. Deprescribing is not commonly taught, and maybe it’s getting better for pharmacists, but not for doctors. We don’t have many guidelines, so they often don’t know how to start the deprescribing process. And because of that, clinicians are afraid of stopping medications or reducing doses, hence, we see adverse drug events.” [Clinical Pharmacists] |
| *Informal caregiver support* | “In care, family can become the critical competent. But you’ve got to take into account that families are willing to take that level of responsibility. Some families feel that they want that, they want their loved one out of hospital, and they recognise that, actually, home is safer than hospital. Other families will be like, no, hospital is where they should be. This is not my job or responsibility.” [Occupational Therapist]  “It could be care home beds or somewhere where you can admit the person for a short period of time so that they can get physical care and the family can get that respite. [General Medicine Consultant] |
| *New professional roles* | “We’ve introduced and are piloting a complex discharge planner. She is a prescriber and can prescribe and write up their [patient] discharge letters. And from the minute complex patients come into the hospital, she starts planning for their discharge.” [Hospital Clinical Nurse Manager] |
| **Technologies**  *Shared information systems* | “…I’ve ended up phoning a GP to ask a question for 3-4 hours, you know. And you end up using a doctor’s time, and I know GPs are always extremely busy, trying to call them when I’m free, hoping they can call me back when they’re free to talk to each other. And then you get them and all you want to know is, “Why did you stop that antidepressant?” Whereas, if I could have seen that on their records, that would have saved everyone’s time.’ [Old Age Psychiatrist] |
| **Information and research**  *Risk stratification using routinely collected data* | “So I think risk of hospitalisation and life expectancy type risk stratification tools will be really helpful, particularly when we're trying to practice realistic medicine. You work out this patient has stage 4 kidney disease and a few other things, but at the moment, they're quite well, so how aggressive should I be in trying to manage and reverse these things? Or is their life expectancy going to be 10 months, and should we focus on what we need to do to make those the best 10 months that they can be?” [General Medicine Consultant] |
| **MACRO LEVEL** | |
| **Service delivery**  *Service availability & access* | “We’re seeing more people that are coming to the front door who have been waiting for care for a long time. If that care had been available in the community, they would have been able to manage at home and manage their medical condition and avoid presentation. But if they’re at home and struggling to feed themselves and struggling to tend to their personal care, then things tend to break down.” [Hospital Allied Health Professional] |
| **Workforce**  *Workforce education and planning* | “We still get letters from secondary care that give harmful advice to GPs. This happens on an almost everyday basis. It’s about parts of the system not being frailty aware and only seeing things through the lens of a single condition. For example, if the patient who had a stroke is 85y and has mild systolic hypertension, has a history of falls and has fallen and fractures in the past, and has documented postural hypotension then we should not be getting a letter from secondary care saying note to GP – “Make sure this patient’s blood pressure is below 130/80.” [General Practitioner]  “So I can say that the hospital system just now is under a lot of pressure because of short-staffing everywhere. Now and very often the first people to get pulled are specialist nurses. They get pulled into the wards to deal with the day-to-day running of the place rather than focusing on what they’ve dedicated themselves to.” [Hospital Clinical Nurse Manager] |
| **Financing** *Financial systems for health and social care* | I think improving care quality comes down to education and engagement. It is to engage GPs in such a way that they recognise that it's a practice priority to improve the identification and early targeting of frail patients. That needs to be incentivised both with money and with resources and with training. It needs to be part of a system-wide adoption where everybody is on the same page so that everybody realises that frailty is not going away. [Older People’s Community Service Manager]  “We are really stretched and desperate for the beds, but they’re [social care] really short-staffed and resourced and don’t have the capacity and the staff to go in and do the care, so we’re waiting for packages of care while pressured for the beds.” [Clinical Nurse Manager] |
